# Response of Natural Cyanobacteria and Algae Assemblages to a Nutrient Pulse and Elevated Temperature

**DOI:** 10.3389/fmicb.2018.01851

**Published:** 2018-08-13

**Authors:** Miquel Lürling, Mariana Mendes e Mello, Frank van Oosterhout, Lisette de Senerpont Domis, Marcelo M. Marinho

**Affiliations:** ^1^Aquatic Ecology and Water Quality Management Group, Department of Environmental Sciences, Wageningen University & Research, Wageningen, Netherlands; ^2^Department of Aquatic Ecology, Netherlands Institute of Ecology (NIOO-KNAW), Wageningen, Netherlands; ^3^Department of Biology, Federal University of Juiz de Fora, Juiz de Fora, Brazil; ^4^Laboratory of Ecology and Physiology of Phytoplankton, Department of Plant Biology, Rio de Janeiro State University, Rio de Janeiro, Brazil

**Keywords:** blooms, climate change, competition, global warming, optimum growth

## Abstract

Eutrophication (nutrient over-enrichment) is the primary worldwide water quality issue often leading to nuisance cyanobacterial blooms. Climate change is predicted to cause further rise of cyanobacteria blooms as cyanobacteria can have a competitive advantage at elevated temperatures. We tested the hypothesis that simultaneous rise in nutrients and temperature will promote cyanobacteria more than a single increase in one of the two drivers. To this end, controlled experiments were run with seston from 39 different urban water bodies varying in trophic state from mesotrophic to hypertrophic. These experiments were carried out at two different temperatures, 20°C (ambient) and 25°C (warming scenario) with or without the addition of a surplus of nutrients (eutrophication scenario). To facilitate comparisons, we quantified the effect size of the different treatments, using cyanobacterial and algal chlorophyll a concentrations as a response variable. Cyanobacterial and algal chlorophyll a concentrations were determined with a PHYTO-PAM phytoplankton analyzer. Warming caused an 18% increase in cyanobacterial chlorophyll-*a*, while algal chlorophyll-*a* concentrations were on average 8% higher at 25°C than at 20°C. A nutrient pulse had a much stronger effect on chlorophyll-*a* concentrations than warming. Cyanobacterial chlorophyll-*a* concentrations in nutrient enriched incubations at 20 or 25°C were similar and 9 times higher than in the incubations without nutrient pulse. Likewise, algal chlorophyll-*a* concentrations were 6 times higher. The results of this study confirm that warming alone yields marginally higher cyanobacteria chlorophyll-*a* concentrations, yet that a pulse of additional nutrients is boosting blooms. The responses of seston originating from mesotrophic waters seemed less strong than those from eutrophic waters, which indicates that nutrient control strategies –catchment as well as in-system measures– could increase the resilience of surface waters to the negative effects of climate change.

## Introduction

Over-enrichment of surface waters by nutrients from agricultural, industrial and urban discharges – eutrophication – is a major threat to the quality and beneficial use of freshwater resources (Paerl and Paul, 2012). Eutrophication has become a worldwide water quality issue (Smith and Schindler, 2009). Scientists have recognized eutrophication as the most important water quality problem and threat to the quality of freshwater resources for decades to come (Downing, 2014). Under eutrophication the ecosystem state of water body is often characterized by a turbid water column dominated by phytoplankton, while under less nutrient rich conditions water may be clear with abundant submerged macrophytes (Scheffer and van Nes, 2007). In the eutrophic state, especially cyanobacteria can reach high densities through excessive cyanobacterial growth and accumulations at the water surface (Watson et al., 1997; Smith et al., 1999; Paerl et al., 2011). These cyanobacterial blooms and surface scums are a threat to human and animal health as many cyanobacteria can produce a suite of potent toxins (Codd et al., 2005; Dittmann and Wiegand, 2006). Globally, the incidence and intensity of such cyanobacterial blooms are on the rise (de Figueiredo et al., 2004; Paerl et al., 2011; O’Neil et al., 2012).

Main drivers of the intensification of cyanobacterial blooms are inefficient waste water treatment (van Loosdrecht and Brdjanovic, 2014), increased use of fertilizers (Bennett et al., 2001) and climate change (Paerl and Huisman, 2008; Moss et al., 2011) – all three consequences of a growing world population. Direct warming effects on cyanobacteria growth rates are predicted to favor cyanobacteria over freshwater eukaryotic phytoplankton at elevated temperatures (Paerl and Huisman, 2008, 2009; Paerl and Paul, 2012). Furthermore, warming-enhanced nutrient loading by temperature-mediated higher phosphorus (P) release from the sediment (Jeppesen et al., 2009) and by increasing the rate of mineralization in catchment soils (Moss et al., 2011) is also predicted to intensify the symptoms of eutrophication (Moss et al., 2011; O’Neil et al., 2012; Paerl and Paul, 2012; De Senerpont Domis et al., 2013). Associated with warming is higher winter rainfall in northern temperate regions that will increase P loading from land to surface water (Jeppesen et al., 2009) and short, intense storms that increase soil erosion and likely intensify run-off and influx of nutrients therewith increasing biomass of potentially toxin-producing cyanobacteria (Trolle et al., 2016). Particularly, projected short intense summer storms during periods of droughts (Bates et al., 2008) may fuel receiving waters with a pulse of nutrients during the growing season thus further promoting cyanobacteria growth (Elliott, 2012a,b). This is corroborated in a recent experiment with water from a eutrophic urban pond, where adding a pulse of nutrients and warming boosted cyanobacterial biomass (Lürling et al., 2017). The effect of warming became evident when nutrients were added simultaneously, while nutrients had a positive impact on cyanobacteria biomass increase even without warming (Lürling et al., 2017). Likewise, modeling predicted that dominance of cyanobacteria was greatly enhanced under higher nutrient load scenarios and warming, but less so under lower nutrient load scenarios (Elliott, 2012b). In a multi-lake analysis, nutrients also proved to be a more powerful predictor of cyanobacterial biomass than temperature, while in more eutrophic lakes cyanobacteria appeared more sensitive to the interaction of nutrients and temperature (Rigosi et al., 2014). Using an experimental time series approach, De Senerpont Domis et al. (2014) were able to distinguish the effect of warming and nutrient loading on total phytoplankton biomass build-up and growth rates. While growth rates were only affected by temperature, biomass build-up was affected by both warming and increased nutrient loading, potentially due to higher nutrient use efficiency at higher temperatures (De Senerpont Domis et al., 2014). In general, low-nutrient water bodies will probably be more resilient to the expected adverse effects of warming than eutrophic waters and are unlikely to build cyanobacterial blooms under warmer conditions (Brookes and Carey, 2011).

In this study we aimed at gaining insight on the rapid response of urban waters to a pulse of nutrients, as predicted from short intense summer storms during periods of droughts (Bates et al., 2008). Using water from 39 mesotrophic to hypertrophic lakes and ponds, we tested the hypotheses that; (1) warming will promote cyanobacteria, (2) lower-nutrient waters are more resilient to a pulse of nutrients, (3) warming is stimulating cyanobacteria in eutrophic waters and (4) the response depends on the presence of the most dominant cyanobacteria in the starting water. As we were interested in the response of the phytoplankton community in presence of grazers and competitors, we used unfiltered water. To this end, we incubated aliquots at 20°C (considered normal temperature) or 25°C (warming scenario) without or with nutrient addition (eutrophication pulse scenario), where after the response of cyanobacteria- and eukaryote algal chlorophyll-*a* was determined.

## Materials and Methods

### Sampling of Surface Waters

A total of 39 freshwaters were sampled in the Netherlands during summer 2010 (**Supplementary Table S1**). On site, dissolved oxygen concentration and saturation (Oxyguard Handy Polaris, OxyGuard International A/S, Farum, Denmark), conductivity (WTW-Cond 330i; WTW GmbH & Co., KG, Weilheim, Germany), pH (WTW-pH320), water temperature and Secchi-depth were measured. Four liter integrated water samples were taken with a sampling tube (diameter 5 cm, length 1 m) and were brought to the laboratory. Total- and cyanobacterial chlorophyll-a concentrations were measured using a PHYTO-PAM phytoplankton analyzer (Heinz Walz GmbH, Effeltrich, Germany) that was calibrated against the Dutch standard for chlorophyll-a analysis (NNI, 2011 – ISO 10260), which is a hot ethanol extraction based on Moed and Hallegraeff (1978). The application of four different excitation wavelengths in the PHYTO-PAM allow for a separation between cyanobacteria and eukaryote phytoplankton in the water (Kobolski and Schreiber, 1995; **Supplementary Figure S1**). We refer to chlorophyll-a concentrations determined in the blue channel as cyanobacterial chlorophyll-a and the sum of the green and brown channel as eukaryote algae chlorophyll-a (Schreiber, 1998). Total chlorophyll-a is the sum of all three channels. Previous work has shown that the PHYTO-PAM was able to quantify correctly the three different major pigment-based plankton groups in field samples from Lake Ontario (Beecraft et al., 2017). Our own work support these findings and shows that the PHYTO-PAM is able to distinguish major phytoplankton groups (**Supplementary Figure S1**) in mixtures of known composition (**Supplementary Figure S2**), gives good estimates of the chlorophyll-a concentrations compared to the Dutch standard (**Supplementary Figure S3**) and correlates well with cell counts (**Supplementary Figure S4**).

The measured cyanobacterial chlorophyll-a concentrations were used to classify the waters in risk categories according to the Dutch Cyanobacteria Protocol (Ibelings et al., 2012, 2014). The “no risk” category includes waters with a cyanobacterial chlorophyll-a concentration <12.5 μg l^−1^; the “low risk” or Alert Level 1 is used for waters with a cyanobacterial chlorophyll-a concentration between 12.5 and 75 μg l^−1^; while the “health risk” or Alert Level 2 is designated to waters with a cyanobacterial chlorophyll-a concentration of >75 μg l^−1^.

Turbidity was measured with a Hach 2100P Turbidity meter (Hach Nederland, Tiel, Netherlands). Total phosphorus (TP) and total nitrogen (TN) concentrations were determined in unfiltered water samples using a Skalar SAN+ segmented flow analyzer (Skalar Analytical BV, Breda, Netherlands) following the Dutch standard protocols (NNI, 1986, 1990). Glass-fiber filtered (Whatman GF/C, Whatman International Ltd., Maidstone, United Kingdom) samples were analyzed for dissolved inorganic nitrogen (DIN, i.e., ammonium and nitrate plus nitrite) and phosphate concentrations (Skalar SAN+ segmented flow analyzer, NNI, 1986, 1990, 1997).

Since our response variable was phytoplankton chlorophyll-a, we chose chlorophyll-a as the metric to determine the trophic state of the waters. This trophic status of the sampled water bodies was determined using the chlorophyll-a based trophic state index (TSI) developed by Carlson (1977). Potential N- or P limitation of phytoplankton growth of the sampled waters was inferred from DIN:SRP and from TN:TP ratios (Kosten et al., 2009). When water TN:TP ratios were below 20 (molar based), the water was considered N limited, while ratios above 38 were considered indicative of P limitation; ratios between DIN and SRP concentrations below 13 (molar based) were considered indicative for N-limitation and those above 50 for P-limitation (Kosten et al., 2009).

### Experiments With Collected Surface Water

Water from each location was used in experiments to test the effect of elevated temperature (warming), nutrient addition (eutrophication) and both (warming + eutrophication) on phytoplankton biomass and composition of major groups, i.e., cyanobacteria and eukaryote chlorophyll-a as determined by a PHYTO-PAM (**Supplementary Material**). Hereto, aliquots of 50 ml were transferred to 100 ml Erlenmeyer flasks that were closed with a cellulose plug. A full-factorial design was used, with temperature (20 and 25°C) and nutrient addition (presence or absence), as blocking factors. Experiments were carried out in triplicate. Water from each location was added to 12 Erlenmeyer flasks of which 6 received nitrogen (14 mg N l^−1^ as NaNO_3_) and phosphorus (1.4 mg P l^−1^ as K_2_HPO_4_). The nutrients were added as a 50 μl spike from a NaNO_3_ (14 g N l^−1^) stock and 50 μl from a K_2_HPO_4_ stock (1.4 g P l^−1^). The flasks were incubated at either 20 or 25°C for 1 week in a Sanyo Gallenkamp incubator. These temperatures were based on water temperatures measured during several summer field campaigns that revealed 20°C as a common average summer water temperature and 25°C as typical warm summer conditions (**Supplementary Figure S5**). In both incubators, light was provided from above by fluorescent tubes at 140 μmol photons m^−2^ s^−1^ in 18:6 h light:dark cycles. Flasks were randomly positioned in the incubator and shaken continuously at 75 rpm.

At the start and end of the experiment, cyanobacterial and eukaryote algae chlorophyll-a concentrations were measured using the PHYTO-PAM phytoplankton analyzer (PHYTO-ED, system II version; **Supplementary Material**). The chlorophyll-a concentration was used as endpoint, because chlorophyll-a is considered a reliable measure of the response to eutrophication (Lambou et al., 1983; Kasprzak et al., 2008). As we were interested in the response to additional warming and nutrients, we defined the incubations at 20°C (without a pulse of nutrients added) as our control, which is corroborated by the mean water temperature during sampling (**Supplementary Table S3**).

### Data Analysis

Water quality variables were compared by Pearson Product Moment Correlation using the program SigmaPlot (version 13.0; Systat Software Inc., San Jose, CA, United States).

As the waters differed broadly in initial and final chlorophyll-*a* concentrations, in the treatments effects were compared using log response ratios (RRs). The RR_T_ is the natural-log proportional change in the means of the response variable in treatment T (warming [RR_25°C_] or eutrophication [RR_20°C__+N+P_] or both [RR_25°C__+N+P_]) and control C (unenriched, 20°C). RRs are commonly used as effect size metric in ecological research (Hedges et al., 1999; Lajeunesse, 2011) and has also been applied in a meta-analysis on the response of phytoplankton to nutrient enrichment (Elser et al., 2007).

RRs for cyanobacteria and for eukaryote phytoplankton were compared by one-way ANOVAs in the tool pack SigmaPlot 13. RRs were also grouped in classes based on the most dominant cyanobacteria (genus level) present at start and analyzed by a two-way ANOVA with genus and treatments as factors. To detect differences between groups, Holm–Sidak *post hoc* comparisons were carried out. Prior to analysis, assumptions of ANOVA were tested using a Shapiro–Wilk test for normality, whereas homogeneity of variance was tested by Levene’s Equal Variance Test. In case one of the assumptions was violated, we proceeded with a non-parametric analysis of variance i.e., a Kruskal–Wallis One Way Analysis of Variance on Ranks followed by a Tukey test or Dunn’s method in case of unequal sample sizes to distinguish among differences.

In addition to analyzing chlorophyll-a concentrations as endpoints, we also determined cyanobacterial- and eukaryote phytoplankton growth rates for each experimental unit. To this end, we used the cyanobacterial and eukaryote phytoplankton chlorophyll-a concentrations (CHL) that were measured at the start and end of the experiment and assuming exponential growth over the 7 days incubation period. Hence, growth rates (μ) were calculated as μ = ln(CHL_end_/CHL_start_)/7. Growth rates for cyanobacteria as well as growth rates for eukaryote phytoplankton were compared by two-way ANOVAs with temperature and nutrients addition treatment as fixed factors in SPSS (version 22). Normality was checked by visual inspection of Q-Q plots and equal variances by Levene’s test.

## Results

### The Sampled Surface Waters

**FIGURE 1:**
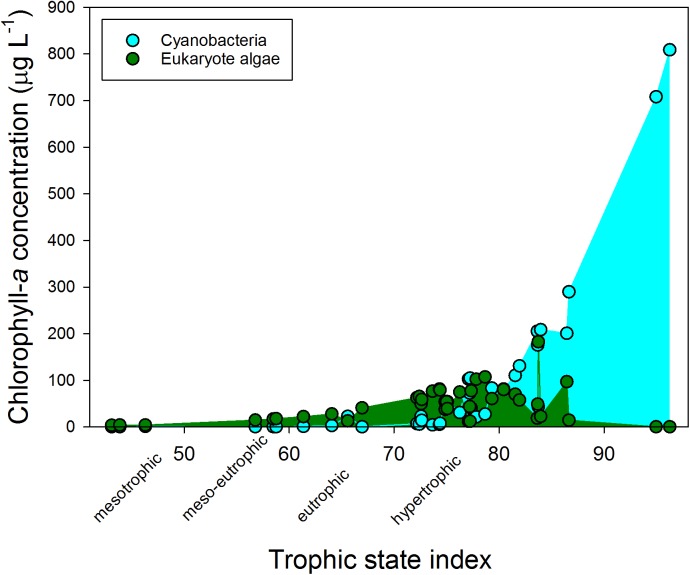
Cyanobacterial (blue) and eukaryote phytoplankton (green) chlorophyll-a concentrations (μg l^−1^) in fresh waters sampled in the Netherlands in summer 2010 and that were used in the experiments. The trophic state index is calculated from the total chlorophyll-a concentrations according to Carlson (1977).

The water bodies were classified as mesotrophic to hypertrophic on chlorophyll-a based trophic state index (TSI) (**Figure 1**). The hypertrophic waters showed a clear dominance of cyanobacteria, while at lower trophic states eukaryote algae prevailed. The cyanobacterial chlorophyll-a concentration in the various water bodies was positively correlated with turbidity (*r* = 0.877; *p* < 0.001), total chlorophyll-a concentrations (*r* = 0.975; *p* < 0.001) and with ammonium concentrations (*r* = 0.365; *p* = 0.022), but not with water temperature on site during sampling (*r* = 0.052; *p* = 0.754) or any of the other water quality variables (**Supplementary Table S2**). Likewise, total chlorophyll-a concentration was correlated with turbidity (*r* = 0.869; *p* < 0.001), and with ammonium concentrations (*r* = 0.327; *p* = 0.042) (**Supplementary Table S2**). The mean water temperature at the sampling locations was 19.9°C (**Supplementary Table S3**).

According to the Dutch Cyanobacteria Protocol, 36% of the urban waters included in this study was at the moment of sampling in the “no risk” category (cyanobacterial chlorophyll-a <12.5 μg l^−1^), 31% was in the Alert Level 1 (small health risks; cyanobacterial chlorophyll-a 12.5 – 75 μg l^−1^) and 33% was in Alert Level 2 (elevated health risks; cyanobacterial chlorophyll-a >75 μg l^−1^).

Based on the TN:TP ratios and the DIN:SRP ratios, most waters were classified as being N-limited (**Supplementary Figure S6**).

### The Effect of Warming

**FIGURE 2:**
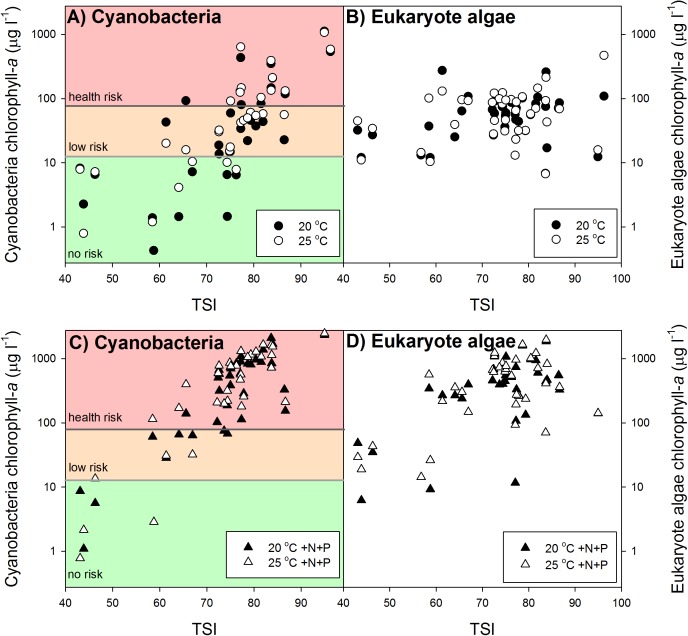
Mean cyanobacterial (left panels **A,C**) and eukaryote phytoplankton (right panels **B,D**) chlorophyll-a concentrations (μg l^−1^) in samples from surface waters varying in trophic state (TSI) after one week incubation at 20°C (filled circles), 25°C (open circles), 20°C with additional nutrients (N and P) added (20°C +N+P; filled triangles) and at 25°C with additional nutrients added (25°C +N+P; open triangles). In the cyanobacteria panels the colored backgrounds indicate the different chlorophyll-a based risk levels according to the Dutch cyanobacteria protocol (Ibelings et al., 2012). Green = no risk (<12.5 μg l^−1^), orange = low risk (12.5–75 μg l^−1^), and red = health risk (>75 μg l^−1^).

Our results revealed no clear overall warming effect (**Figures 2A,B**). In aliquots coming from lower trophic states cyanobacterial chlorophyll-*a* concentrations were not enhanced at 25°C or even lower than those at 20°C. However, in aliquots from higher trophic states cyanobacterial chlorophyll-a concentrations were higher in the warming treatments than in the ambient treatments (**Figure 2A**). Nonetheless, warming did not change health risk according to the Dutch Cyanobacterial Protocol, which is primarily cyanobacterial biomass based and not build on cyanotoxin analysis. In the 20°C as well as in the 25°C incubations 38% of the waters were in the “no risk” category, 33% was in the “low risk” category (Alert Level 1) and 28% was in Alert Level 2 (**Figure 2A**).

**FIGURE 3:**
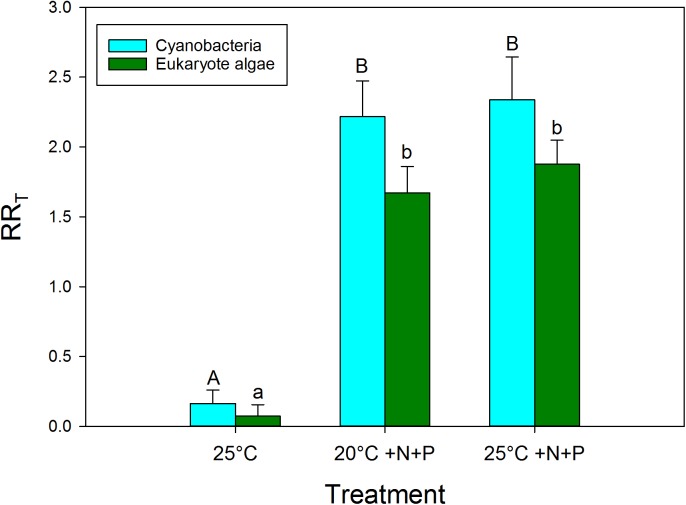
Response ratios (RR_T_), which is the natural logarithm of the quotient of chlorophyll-a concentration (μg l^−1^) in a nutrient addition or warming treatment and control (20°C), for cyanobacterial chlorophyll-a concentrations (blue) and eukaryote phytoplankton chlorophyll-a concentrations (green) in warmed (25°C), nutrient enriched (20°C +N+P) and both warmed + nutrient enriched (25°C +N+P) incubations. Error bars indicate 1 SD. Different letters above the bars, indicate for cyanobacteria and for eukaryote phytoplankton significant difference between groups.

Based on the response ratios (RR_T_) warming had a positive effect on cyanobacteria chlorophyll-a concentrations (**Figure 3**). The RR_25°C_ for cyanobacterial chlorophyll-a was on average 0.165, which equals an increase of 18% in cyanobacterial chlorophyll-a compared to the incubations at 20°C. In seven experiments, no RR_25°C_ could be determined, because the cyanobacterial chlorophyll-a concentration was below detection limit (0.1 μg/L, 5 in the 20°C treatments, and 2 in the 25°C treatments). The vast majority of the experiments, 24 cases, showed positive RR_25°C_ with a mean of 0.38, which equals 46% higher cyanobacterial chlorophyll-a concentrations at 25°C compared to 20°C. In eight cases the RR_25°C_ was negative meaning a decrease in cyanobacterial chlorophyll-a when cultured at 25°C compared to 20°C. Four of these cases involved mesotrophic waters that initially had low cyanobacterial chlorophyll-a, while in the other four cases the aliquots from hypertrophic waters were dominated by *Aphanizomenon* sp. and/or *Dolichospermum* sp.

To evaluate the difference in response of waters of different trophic state, the dataset was split into two groups, one below TSI of 70 and one above. This yielded a RR_25°C_ of −0.25 for the group with TSI <70 and a RR_25°C_ of 0.30 for the group with TSI >70. Hence, the lower trophic state waters in general had 22% less cyanobacterial chlorophyll-a at 25°C than at 20°C, while the higher trophic state waters on average had 35% more cyanobacterial chlorophyll-a 25°C compared to 20°C (**Figure 2A**).

**FIGURE 4:**
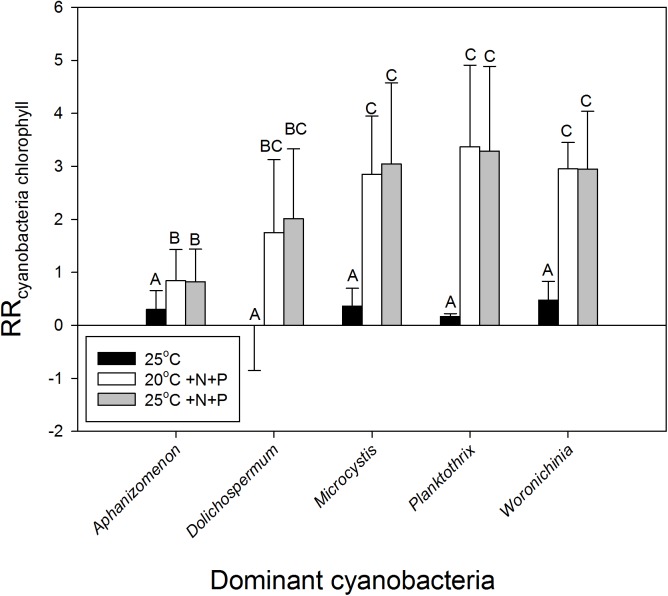
Response ratios - natural logarithm of the quotient of cyanobacterial chlorophyll-a concentrations (μg l^−1^) in warmed (25°C, black bars), nutrient enriched (20°C +N+P, open bars), or warmed + nutrient enriched (25°C +N+P, gray bars) incubations and the control incubations (20°C) – for waters in which at start of the experiments different cyanobacteria were dominant (genus level). Error bars indicate 1 SD, while similar letters indicate homogeneous groups (Tukey test).

To evaluate the difference in response of waters in light of the dominant cyanobacterial species present,, the response ratios were categorized into the presence of the most dominant cyanobacteria in the starting water. This yielded, however, no difference in response to warming (*H*_4_ = 2.767; *p* = 0.598) based on a Kruskal–Wallis One Way Analysis of Variance on Ranks, which was run because normality (Shapiro–Wilk) had failed (*p* < 0.050). Nonetheless, on average the response ratios in waters dominated by *Aphanizomenon* sp., *Microcystis* sp. or *Woronichinia naegeliana* were 0.30, 0.36, and 0.48, respectively, implying an increase in cyanobacterial chlorophyll-*a* of around 35, 44, and 61% in the 25°C incubations compared to the 20°C incubations (**Figure 4**). When we only looked at the effect of warming (without nutrient addition), response ratios in waters dominated by *Planktothrix* sp. were on average 0.17 (18% increase), whereas those in water dominated by *Dolichospermum* sp. showed on average no difference with the 20°C incubations (**Figure 4**).

The effect of warming on eukaryote phytoplankton was less than on cyanobacteria (**Figure 3**). The RR_25°C_ for algal chlorophyll-a was on average 0.075, which implies on average 8% more algae at 25°C than at 20°C. In 23 cases the RR_25°C_ was positive and algae increased under warming. However, in 16 cases RR_25°C_ appeared negative indicating algal chlorophyll-a decreased. As diatoms might be more susceptible than chlorophytes to warming (Butterwick et al., 2005), we checked the original data as the PHYTO-PAM distinguishes three major groups, i.e., “blue” (cyanobacteria), “green” (mostly chlorophytes) and “brown” (mostly diatoms). However, in 15 out of the 16 cases of a decline in algal chlorophyll-a at elevated temperature, the decrease was due to a decline in the “green” algal chlorophyll-a, while in only one water, a mesotrophic pond, the decline was caused by a decrease in diatoms.

### The Effect of Nutrient Pulse

Nutrient enrichment had a much stronger effect on cyanobacteria biomass than warming (**Figures 2**, **3**). RR_20°C__+N+P_ and RR_25°C__+N+P_ for cyanobacterial chlorophyll-a was on average 2.2, indicating an increase of about 900% compared to the incubations at 20°C (RR_T_ = ln [T/C], hence T/C = e^RR^_T_). Only in two mesotrophic systems a negative RR_T_ remained. A two-way ANOVA on RR_T_ for cyanobacterial chlorophyll-a in categories of dominant cyanobacteria in the starting water indicated a significant genus effect (*F*_4,74_ = 5.186; *p* = 0.001), a significant treatment effect (*F*_2,74_ = 29.717; *p* < 0.001) and no genus x treatment interaction (*F*_8,74_ = 1.023; *p* = 0. 429). Tukey’s test revealed that for each dominant cyanobacterial genus, RR_25°C_ was significantly lower than RR_20°C+N+P_ and RR_25°C+N+P_ (**Figure 4**). Moreover, in *Aphanizomenon* sp. dominated waters RR_20°C__+N+P_ and RR_25°C__+NP_ were significantly lower than those in *Microcystis* sp.*, Planktothrix* sp. or *Woronichinia naegeliana* dominated waters (**Figure 4**).

The pulse of nutrients pushed a substantial part of the “no risk” and “low risk” waters to the “health risk” level according to the Dutch Cyanobacterial Protocol (**Figure 2C**). In the 20°C +N+P incubations only 13% of the waters remained in the “no risk” category, 13% in the “low risk” category and 74% had reached the “health risk” level, while in the 25°C +N+P treatment this was 11, 8, and 81%, respectively (**Figure 2C**).

Similar to the results shown for cyanobacteria, nutrient enrichment had a strong effect on algal chlorophyll-a. RR_20°C__+N+P_ and RR_25°C__+N+P_ for algal chlorophyll-a was on average 1.65 and 1.82 indicating 5.2 and 6.1 times higher chlorophyll-a concentrations compared to the unenriched incubations at 20°C (**Figure 3**). In four cases RR_20°C__+N+P_ was negative and in two cases RR_25°C__+N+P_ was negative, which means that algal chlorophyll-a concentrations were lower in these treatments than in the 20°C incubations without nutrients added.

### The Effects of Warming, Nutrient Pulse and Both

A Kruskal–Wallis One Way Analysis of Variance on Ranks showed significant differences (*H*_2_ = 36.431; *p* < 0.001) between the RR_T_’s for cyanobacterial chlorophyll-a. Tukey *post hoc* comparison revealed that RR_25°C_ was significantly lower than RR_20°C__+N+P_ and RR_25°C__+N+P_, but that there was no difference between RR_20°C__+N+P_ and RR_25°C__+N+P_ (**Figure 3**). Where in both the 20°C treatment as well as in the 25°C treatment similar shares were in the “no risk (38%), “low risk” (33%) and “health risk” (28%) category (**Figure 2A**), the combined effect of warming and a pulse of nutrients seemed to further aggravate the hazard, based on cyanobacterial chlorophyll-a concentrations. In the 20°C +N+P treatment 74% was in the “health risk” category, which was further increased to 81% of the waters in the 25°C +N+P treatment (**Figure 2C**).

The Kruskal–Wallis One Way Analysis of Variance on Ranks on the RR_T_’s for eukaryote algae chlorophyll-a also indicated significant differences (*H*_2_ = 47.972; *p* < 0.001). Dunn’s *post hoc* comparison revealed that RR_25°C_ was significantly lower than RR_20°C__+N+P_ and RR_25°C__+N+P_, but that there was no difference between RR_20°C__+N+P_ and RR_25°C__+N+P_ (**Figure 3**).

**FIGURE 5:**
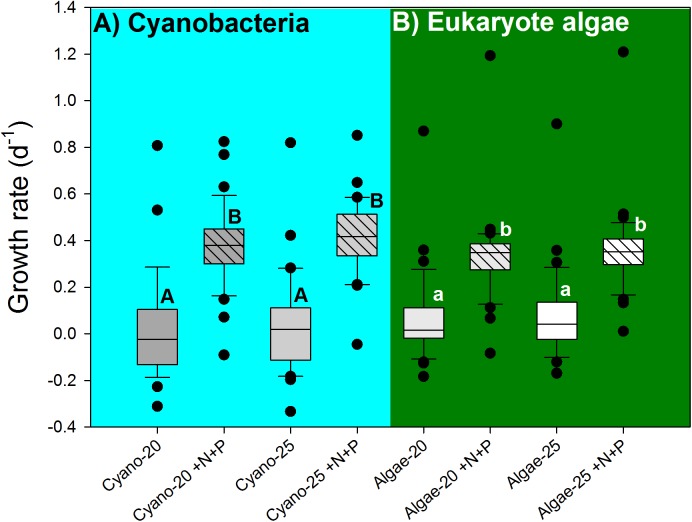
Cyanobacterial growth rates **(A)**, based on chlorophyll-a concentrations (μg l^−1^) and eukaryote algae growth rates **(B)** in water samples from 39 urban ponds and lakes incubated for 7 days at 20°C or 25°C without extra nutrients or with a pulse of N and P (+N+P). The boxes indicate the 25th – 75th percentiles, the line the median, the whiskers the 10th and 90th percentile and the dots each outlier. Similar letters (A,B for cyanobacteria and a,b for eukaryote algae) indicate homogeneous groups that are not different from each other.

The cyanobacterial growth rates were not influenced by temperature (*F*_1,130_ = 0.273; *p* = 0.602), but significantly elevated by the nutrient pulse (*F*_1,130_ = 86.880; *p* < 0.001), while the two-way ANOVA indicated no temperature x nutrient interaction (*F*_1,130_ = 0.006; *p* = 0.939). Hence, there were two homogenous groups: (1) the 20°C and 25°C treatments, and (2) the 20°C +N+P and 25°C +N+P treatments (**Figure 5A**).

Likewise, eukaryote algae growth rates were not influenced by temperature (*F*_1,147_ = 0.621; *p* = 0.432), but significantly elevated by the nutrient pulse (*F*_1,147_ = 145.347; *p* < 0.001), while the two-way ANOVA indicted no temperature x nutrient interaction (*F*_1,147_ = 0.072; *p* = 0.789). Hence, two homogenous groups were detected: (1) the 20°C and 25°C treatments, and (2) the 20°C +N+P and 25°C +N+P treatments (**Figure 5B**).

**FIGURE 6:**
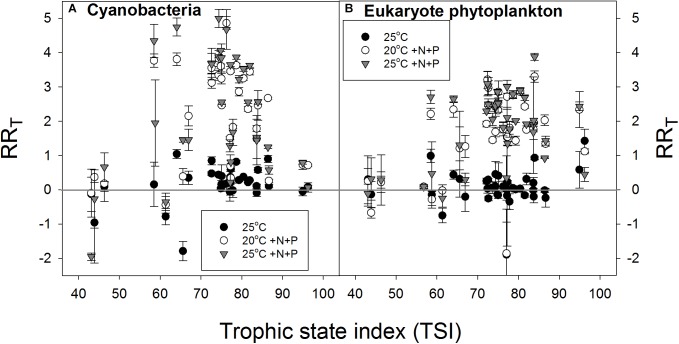
Response ratios (RR_T_), which is the natural logarithm of the quotient of chlorophyll-a concentration (μg l^−1^) in a treatment and control (20°C), for cyanobacterial chlorophyll-a concentrations **(A)** and eukaryote phytoplankton chlorophyll-a concentrations **(B)** in warmed (25°C, filled circles), nutrient enriched (20°C +N+P, open circles) and both warmed + nutrient enriched (25°C +N+P, gray triangles) incubations.

In support of the hypothesis that lower-nutrient waters are more resilient to a pulse of nutrients, the RR_T_ for cyanobacterial chlorophyll-a seemed lowest in waters with the lowest TSI and in waters with the highest TSI expressing a bell shaped response (**Figure 6A**). The RR_T_ for eukaryote chlorophyll-a seemed also lowest in samples from waters with the lowest TSI, but remained elevated at the highest TSI’s (**Figure 6B**).

## Discussion

The vast majority - 37 out of 39 - of the sampled waters were urban ponds and of the two remaining sites, one was in an urban harbor and the other just outside a village on a 6150 ha lake area. Large lakes (>50 ha) are under regular monitoring programs such as demanded within the Water Framework Directive (European Union, 2000). However, water authorities have a blind spot concerning smaller water bodies such as ponds (Waajen et al., 2014) despite waters <50 ha dominating the total global area covered by inland waters (Downing et al., 2006; Verpoorter et al., 2014). Waters in urbanized areas contribute to the quality of urban life in providing recreational services and amenity to citizens (Bolund and Hunhammar, 1999; Brönmark and Hansson, 2002; Gledhill et al., 2008), but they are also vulnerable to anthropogenic stresses (Waajen et al., 2014). In our selection of sampled waters, based on the Dutch Cyanobacteria Protocol (Ibelings et al., 2012, 2014) only about one third was in the “no risk” category, one third was in the Alert Level 1 (small health risks) and one third was in Alert Level 2 (elevated health risks), underscoring the risks associated with ignoring these frequently used waters in monitoring campaigns.

In this Dutch Cyanobacteria Protocol, the cyanobacterial biomass can be estimated using fluorometry devices specifically targeting cyanobacterial phycocyanin (Ibelings et al., 2012, 2014). Tests of Dutch water authorities revealed that fluorometry was a robust method to estimate cyanobacterial biovolume (van der Oost et al., 2010) and more reliable than cell counts because of in between laboratory variation in counting (van der Oost et al., 2010; Ibelings et al., 2014). The PHYTO-PAM correctly estimated the relative composition of the phytoplankton community in field samples (Beecraft et al., 2017). In our study, PHYTO-PAM derived cyanobacterial and eukaryote algae chlorophyll-a concentrations were in agreement with cell counts (**Supplementary Figure S4**) and the total chlorophyll-a concentration of field samples is estimated well (**Supplementary Figure S3**). Similarly, PHYTO-PAM gave a good proxy of the chlorophyll-a concentrations in mixtures of laboratory phytoplankton cultures (Jakob et al., 2005), which was confirmed in our tests (**Supplementary Figure S2**). Despite several factors that can produce noise in chlorophyll-a fluorescence based phytoplankton biomass estimation and major pigment group community composition (Catherine et al., 2012), the PHYTO-PAM appears robust enough to detect changes in the share of cyanobacterial and non-cyanobacterial chlorophyll-a concentrations. Distinguishing between major pigment groups of eukaryote phytoplankton (diatoms and chlorophytes), however, might be more challenging (**Supplementary Figure S1**).

The results of our short-term experiments provide insight in the rapid response of urban waters to a pulse of nutrients at ambient and predicted future summer temperature. Nutrients wash into water bodies with precipitation based run-off during storms (Hvitved-Jacobsen et al., 1994; Beck and Birch, 2012; Wang et al., 2013). In the region where our ponds were located, run-off to urban ponds during eight precipitation events (12.4 to 41.6 mm rain fall within 24 h) contained total-P concentrations varying between 0.5 and 7.5 mg P l^−1^ (of which 60% -range 55% to 72%- as phosphate) and total-N concentrations between 3.9 and 47.7 mg N l^−1^ (Waajen et al., 2016; **Supplementary Information**). Nutrient concentrations in run-off water may vary due to build-up and wash off processes (Beck and Birch, 2012). In general, nutrient loads are proportional to the length of preceding dry weather periods (Shirasuna et al., 2006) and build-up is usually large enough to ensure continuous wash-off during an entire event (Deletic, 1998). Hence, predicted prolongation of droughts followed by more intense, short summer storms in near future (Bates et al., 2008), will likely increase the nutrient pulses to urban waters that subsequently may suffer from swimming bans and recreational devaluation (Beck and Birch, 2012).

The time-scale of effects from storm run-off nutrient influx may vary from acute effects occurring within days to accumulative effects spread out over years (Hvitved-Jacobsen et al., 1994). In our selection of urban waters the latter is expected to be reflected in the trophic state of the water, while we were interested in the acute effects of a pulse of nutrients, warming and both nutrients plus warming on phytoplankton biomass.

As expected, adding nutrients, on average, boosted algal and cyanobacterial growth, particularly in aliquots from waters in a eutrophic state based on total chlorophyll-a classification. Phytoplankton in samples from waters in lower trophic states, however, showed a less strong response to nutrient additions. Using RR_T_ instead of absolute chlorophyll-a concentrations demonstrated that the lower trophic state waters were capable of absorbing the added nutrients without boosting phytoplankton. Hence, the results of this study are in favor of the hypothesis that lower-nutrient waters are more resilient to a pulse of nutrients than more nutrient enriched waters are. It might be that the added nutrients were taken up by heterotrophic microbial community or that the lower-nutrient waters contained relatively more zooplankton. *In situ* a weakened response to nutrient addition in lower-nutrient waters can be attributed to a complete different plankton community and food web structure (Carpenter and Cottingham, 1997). Where the added nutrients are transferred effectively to zooplankton and to higher trophic levels in resilient, low trophic state waters, they accumulate as phytoplankton biomass in less resilient, high trophic state waters (Carpenter and Cottingham, 1997). We had no oligo- or oligo-mesotrophic waters in our selection, but also in our waters an important role of grazing can be expected. Here, larger bodied grazers in the longer food chains of the resilient, low trophic state waters consume a wider range of phytoplankton species than the smaller bodied grazers in the shorter food chains of less resilient, high trophic state waters (Carpenter and Kitchell, 1993). Consequently, nutrient pulses associated with summer storms are most likely absorbed in resilient, low trophic state waters, whilst fueling cyanobacteria blooms in high trophic state waters.

Based on the RR_T_’s this study yielded no support for the hypothesis that warming stimulates cyanobacteria in waters simultaneously experiencing a pulse of nutrients, as the RR_20°C+N+P_ and RR_25°C+N+P_ were similar and on average 2.17 and 2.24. Nutrient additions had a large impact on presumed health risks according to the Dutch Cyanobacterial Protocol. The 20°C +N+P treatment resulted in 74% of the waters in the “health risk” category, while this was 28% in the 20°C treatment, in the 25°C +N+P treatment this was 81%, compared to 28% in the 25°C treatment.

Thus, a pulse of nutrients reduced the percentage of “no risk” waters from 38% to 11–13% and increased the percentage of “health risk” waters from 28% to 74–81%. Consequently, the urban waters in our study seem vulnerable to pulses of nutrients associated with episodic summer storms, which may in many cases lead to higher cyanobacterial biomass. Although we did not measure cyanotoxins in our study, the elevated cyanobacterial biomass probably will also lead to elevated cyanotoxin concentrations (Davis et al., 2015; Lürling et al., 2017). In many countries alert and action modes apply when cyanotoxin concentrations in recreational waters, predominantly the most common class of microcystins (MC), exceed 10–20 μg l^−1^ (Ibelings et al., 2014). Such concentrations may be quite common in urban waters (Faassen and Lürling, 2013; Waajen et al., 2014) and thus any nutrient pulse in summer will aggravate the potential health risk to citizens that are using the urban waters for recreation. However, as indicated before, water authorities primarily focus on larger water bodies and monitoring of smaller urban waters is virtually absent (Waajen et al., 2014). Therefore, an adequate monitoring program for urban waters should be implemented. This can be done by training the municipal employees that visit those sites regularly in using relatively easy to handle fluorometers, such as the Fluoroprobe (Catherine et al., 2012), and in recognizing blooms and surface scums. Presence of scums or exceeding the chlorophyll-a levels as set in the Dutch cyanobacteria protocol (Ibelings et al., 2012, 2014) should then lead to alerting the respective water authority for further analysis. We recommend to include determination of cyanotoxins, or at least microcystins as the most encountered class of toxins, in the subsequent analysis to get insight in the potential hazard.

In the absence of a nutrient pulse, warming favored cyanobacteria over other phytoplankton as the overall mean cyanobacterial chlorophyll-*a* concentration was 18% higher at 25°C than at 20°C, while for eukaryote algal chlorophyll-*a* concentration this was 8%. Nonetheless, warming did not change health risk according to the Dutch Cyanobacterial Protocol.

Strong increase of the maximum specific growth rate of cyanobacteria with rising temperatures has been proposed as a mechanism giving cyanobacteria a competitive advantage over their eukaryote competitors at elevated temperatures in nutrient-enriched waters (Paerl and Huisman, 2009; Paerl and Paul, 2012). The temperatures used in our study are, however, in the range where no main differences in cyanobacterial or eukaryote algal growth rates are to be expected (Paerl and Huisman, 2009; Paerl and Paul, 2012; Lürling et al., 2013). This is supported by the results of our experiment in which the net cyanobacterial growth rates, based on cyanobacterial chlorophyll-a, were similar at 20°C and 25°C. Importantly, we observed large variability in growth rates, ranging from −0.31 to 0.81 d^−1^ at 20°C and from −0.33 to 0.82 d^−1^ at 25°C.

Growth rates may differ between monocultures and mixed species cultures due to competitive, allelopathic interactions (Marinho et al., 2013; Torres et al., 2016), whereas selective grazing straightforwardly may suppress phytoplankton vulnerable to grazing, whilst facilitating grazing resistant phytoplankton (Ger et al., 2014, 2016). We incubated relatively small volumes of water, which could make the presence of large-bodied grazers a rather stochastic event; however, visual inspections did not yield any large bodied cladocerans present in the experimental units. As our samples came from urban waters that are heavily overstocked with fish (Waajen et al., 2014), fish predation most likely enforced strong control on large cladocera size and density (Peretyatko et al., 2009).

Here, we used a short incubation period of one week for the natural seston reflecting a normal and hot summer period. However, climate change effects are multiple, with the strongest temperature increase being expected during late winter and early spring in temperate regions such as Netherlands (De Senerpont Domis et al., 2007, 2013). In that view, a microcosm experiment with a natural phytoplankton community from a eutrophic lake (devoid of zooplankton) revealed that growth rates of green algae were even slightly higher and more variable than those of cyanobacteria in cold (9–13°C), average (9–19°C) and warm spring warming scenarios (9–25°C, De Senerpont Domis et al., 2007). However, the relative impact of warm spring warming scenarios on cyanobacteria was stronger than on green algae (De Senerpont Domis et al., 2007). Laboratory experiments with 15 pre-cultured phytoplankton species (cyanobacteria, green algae and diatoms) mixed and grown for two weeks at 12, 18, and 24°C yielded no differences in cyanobacterial growth rates, but the relative change in the cyanobacterial biovolume fractions in communities tended to increase with temperature (Schabhüttl et al., 2013). Hence, warming in simulated spring scenarios as well as in short-term experiments show that cyanobacteria may benefit more than their eukaryote competitors.

Water samples used in our current study included the natural plankton community, and thus included both competitive and grazing interactions. In a comparable set-up with water from one urban pond, negative growth rates were found for eukaryote phytoplankton, but not for cyanobacteria indicating that the algae suffered more from grazing by zooplankton than the cyanobacteria did (Lürling et al., 2017). Hence, both the results of that study and our current study point toward a beneficial effect of warming on cyanobacteria, which most likely runs through competitive advantages in nutrient acquisition and grazing resistance (e.g., Tilman et al., 1982; Sterner, 1989a; Yoshida et al., 2007; Ger et al., 2016; Lürling et al., 2017). Since in general biological rates are higher at warmer temperatures, including zooplankton grazing and respiration rates (Moore et al., 1996), also zooplankton-mediated nutrient recycling (Sterner, 1989b) could stimulate grazing resistant cyanobacteria more at elevated temperature.

The overwhelming response to nutrients in our study supports the notion that increased nutrient concentrations are a consistently more important driver of cyanobacterial blooms than warming temperatures, as based on modeling studies, historical data analyses and experimental studies (Brookes and Carey, 2011). A study in the polymictic Müggelsee (Germany) not only revealed that climate-induced changes in the thermal regime rather than a direct temperature effect stimulated cyanobacteria dominance, but also that total phosphorus concentration was the principal force driving cyanobacteria (Wagner and Adrian, 2009). In an analysis of summer snapshot samplings in 1076 lakes in the United States nutrients turned out significantly more important than temperature in promoting chlorophyll-a, cyanobacterial biomass, and cyanobacterial dominance (Rigosi et al., 2014). However, this importance varied with the trophic state of the system, where nutrients were most important in oligotrophic lakes, temperature in mesotrophic lakes, while in eutrophic and hyper-eutrophic lakes a significant interaction between nutrients and temperature was found (Rigosi et al., 2014). Both light limitation as well as the indirect effects of warming on stratification probably resulted in enhanced interactions between nutrients and temperature (Rigosi et al., 2014).

Warming in our study was a water temperature of 25°C compared to 20°C, reflecting, respectively a “ hot” and “ambient” summer. Our experiments were not set out to capture the long term temperature effects in temperate regions, where under eutrophic conditions cyanobacteria usually start to flourish during the warmer periods of the year (Watson et al., 1997). The context of our experiments was to get insight in the rapid response of urban waters to a pulse of nutrients, as predicted from short intense summer storms during periods of droughts (Bates et al., 2008). The outcome of the experiment evidently showed that a pulse of nutrients may promote cyanobacteria and algae strongly as has been predicted for the growing season (Elliott, 2012a,b). Yet, this promotion only occurred in eutrophic waters.

It should, however, be noted that in 36% of the cases in our study cyanobacterial chlorophyll-a concentrations in the 25°C +N+P treatments were lower than in the 20°C +N+P treatments. These were scattered out over the entire range of waters tested, i.e., varying from a TSI of 43 to a TSI of 96, illustrating the response to warming and a nutrient pulse is variable and not clearly linked to the trophic state. In the remaining 64% of the cases the mean cyanobacterial chlorophyll-a concentration was on average 53% higher in the 25°C +N+P treatments than in the 20°C +N+P treatments. A comparable result was obtained in a recent experiment with water from one eutrophic urban pond, where adding a pulse of nutrients and warming boosted cyanobacterial biomass, but warming in itself yielded much less cyanobacterial and algal biomass (Lürling et al., 2017). Clearly, the response to warming and a pulse of nutrients was context dependent. However, the majority of the cases seem to support the reported synergism between temperature and nutrients in eutrophic and hyper-eutrophic lakes (De Senerpont Domis et al., 2014; Rigosi et al., 2014) and the notion that intensifying nutrient inputs and rising temperatures increase eutrophication symptoms (Moss et al., 2011). A contrasting result was found with the invasive cyanobacterium *Cylindrospermopsis raciborskii* where, while warming had no effect on its dominance, it prevailed under low P supply, but was outcompeted by faster growing chlorophytes under high P supply (Ryan et al., 2017). This outcome could likely have been different if the experimental set-up had included the complete species assemblage of interest (Lehman and Sandgren, 1985), such as zooplankton grazers as had been done in our study.

The incubated samples were continuously mixed facilitating determination of a direct warming effect, but *in situ* warming also intensifies thermal stratification, lengthens the period of stratification and lowers viscosity, therewith favoring buoyancy controlled cyanobacteria and the development of surface accumulations of positively buoyant cyanobacteria (Jöhnk et al., 2008; Carey et al., 2012; Paerl and Paul, 2012). These indirect warming effects are likely more important than direct temperature effects on specific growth rates (Wagner and Adrian, 2009; Lürling et al., 2013). Whether cyanobacteria will proliferate to a greater extent or form scums and accumulations more often in a warmer world also depends on meteorological variability promoting or preventing long-lasting thermal stratification (Huber et al., 2012). To yield more insight in such longer term effects, warming scenarios could be employed in highly controlled indoor Limnotron mesocosms (Velthuis et al., 2017).

The overwhelming effect of nutrient pulses in enriched waters, but its absence in less enriched waters, indicates that reducing the trophic state of these urban waters seems a forthright strategy. Therewith the urban waters will not only become more resilient against nutrient pulses (Carpenter and Cottingham, 1997), but also against the negative impacts of predicted climate change (Brookes and Carey, 2011). Similarly, McGowan et al. (2012) pointed out that based on paleolimnological survey in Lake Windermere (United Kingdom) the control of eutrophication is essential in building resilience to future climate change.

Most commonly, physical measures to attenuate run-off are implemented, such as constructed wetlands, porous pavements, retention basins (Goonetilleke et al., 2005). However, in urban areas, these in-catchment measures are confronted with space-limitations that might hamper their effectiveness (Goonetilleke et al., 2005; Huser et al., 2016). High density residential development has been proposed as an option for a relatively smaller footprint (Goonetilleke et al., 2005), but in highly populated countries as the Netherlands this is already commonplace. Consequently, possibilities to reduce the external nutrient load to such urban waters are limited and mostly restricted to behavioral changes in users, such as lowering feeding of birds and fish, limited use of angling bait, reduced fish stocking and removal of leaf litter and dog feces (Waajen et al., 2016). With limited possibilities of reducing external nutrient load, in-lake measures, such as P fixation or removal of nutrient rich sediment, are then the most practical management strategy to mitigate eutrophication in urban waters (Huser et al., 2016). Which measures are most promising to implement will depend on the characteristics of each water and should follow from a proper diagnosis (Lürling et al., 2016; Waajen et al., 2016).

## Author Contributions

ML and MMM conceived the research. ML, FO, and MM performed the sampling. All authors contributed to the analysis and writing of the manuscript.

## Conflict of Interest Statement

The authors declare that the research was conducted in the absence of any commercial or financial relationships that could be construed as a potential conflict of interest.
