# Supplementary material for: Response of Natural Cyanobacteria and Algae Assemblages to a Nutrient Pulse and Elevated Temperature
Source: Front Microbiol. 2018 Aug 13;9:1851. doi: 10.3389/fmicb.2018.01851 (PMC6099115; doi:10.3389/fmicb.2018.01851)
Supplement: Supplementary file 1 [file Data_Sheet_1.DOCX]

**Supplementary Material to Response of natural cyanobacteria and algae assemblages to a nutrient pulse and elevated temperature**

Miquel Lürling, Mariana Mendes e Mello, Frank van Oosterhout, Lisette de Senerpont Domis, Marcelo Manzi Marinho

The supplement contains an overview of several tests to determine the accuracy and capacity of the PHYTO-PAM as a rapid tool to determine cyanobacterial chlorophyll-a concentrations (**Figures S1-S4**). It also contains the mean summer water temperature (ºC) in urban waters in The Netherlands during the period 2006-2012 (**Figure S5**), the TN:TP and DIN:SRP ratios in the samples from 39 surface waters at start of the experiment used to identify N-limited and P-limited conditions (**Figure S6**), an overview of sampled waters, location and sampling date (**Table S1**), correlations of water quality variables (**Table S2**) and an overview of the water quality data (**Table S3**).

**Testing the PHYTO-PAM**

The PHYTO-PAM uses four different excitation wavelengths, provided by LEDs peaking at 470, 535, 620 and 659 nm, which allows a separation between cyanobacteria, green algae and diatoms/dinoflagellates, but also detects other eukaryote phytoplankton in the water. To check the ability of the PHYTO-PAM to distinguish different phytoplankton species four different phytoplankters belonging to the above mentioned major groups were cultured. The cyanobacterium *Microcystis aeruginosa* NIVA-CYA43, the green alga *Scenedesmus obliquus* SAG 276/3a, the diatom *Cyclotella meneghiniana* BCCM/DCG 0500 and the cryptophyte *Cryptomonas sp.* SAG 11.89 were maintained separately in cellulose plug stoppered 250 mL Erlenmeyer flasks containing 125 mL of a modified WC-medium (Lürling and Beekman, 1999) at 25 ˚C under a 14:10 h light/dark cycle at a light intensity of 70 µmol m^-2^ s^-1^. These cultures were regularly transferred into fresh medium.

Placing a 2 mL sample for each species in the measuring cuvette of the PHYTO-PAM confirmed an excellent power to discriminate between cyanobacteria and eukaryote algae (**Figure S1**). The cyanobacterium ended up 100% in the blue channel, the green alga and the diatom fully in the green and brown channel, and only a minor fraction of the fluorescence signal of *Cryptomonas* was identified as cyanobacterial chlorophyll-a (**Figure S1**).

**Figure S1.** The proportion of the chlorophyll-a concentration based on in vivo fluorescence distributed over the blue channel (indicative for cyanobacteria) and the green and brown channel (indicative for eukaryote algae, chlorophytes, diatoms, cryptophytes) for different phytoplankton species.

In a next test, the capacity of the PHYTO-PAM to detect cyanobacteria and green algae and to estimate their individual as well as chlorophyll-a concentrations in known mixtures was examined. To this end, mixtures of the cyanobacterium *Microcystis aeruginosa* NIVA-CYA43 and the green alga *Scenedesmus obliquus* SAG 276/3a were examined.

The chlorophyll-a concentrations in samples consisting of known mixtures yielded excellent chlorophyll estimates for the cyanobacterium, the green alga and of total chlorophyll-a concentrations (**Figure S2**). The chlorophyll estimates for the cyanobacterium were in close match with the expected values: CHL_measured_ = - 31.1368 × 1.1415 CHL_expected_ (r^2^ = 0.979). Likewise, the chlorophyll-a concentrations of *S. obliquus* was reflected in a slope close to 1: CHL_measured_ = 1.3693 × 1.0485 CHL_expected_ (r^2^ = 0.997). Parallel lines analysis revealed that the slopes of both lines were significantly different (*F*_1,24_ = 6.55; *p* = 0.017). The measured total chlorophyll-a concentration was in good agreement with the expected values: CHL_measured_ = 18.9010 × 0.9210 CHL_expected_ (r^2^ = 0.946).

 **Figure S2**. Measured chlorophyll-a concentrations (filled symbols) and expected chlorophyll-a concentrations (open symbols) for the blue channel and green channel of the PHYTO-PAM for various mixtures of the cyanobacterium *Microcystis aeruginosa* (circles) and the green alga *Scenedesmus obliquus* (triangles). Also included are the expected and measured total chlorophyll-a concentrations for the different mixtures (squares).

The PHYTO-PAM was calibrated against the Dutch standard (NNI, 2011), which is a hot ethanol extraction based on Moed and Hallegraeff (1978). A comparison of 115 undiluted field samples in which the total chlorophyll-a concentration was determined both by hot ethanol extraction (CHL_extraction_) and PHYTO-PAM (CHL_PHYTO_PAM_) yielded a very good relation for chlorophyll-a concentrations up to around 1000 µg L^-1^: CHL_PHYTO_PAM_ = - 0.8599 × 0.9152 CHL_extraction_ (r^2^ = 0.937) (**Figure S3**). At higher chlorophyll-a concentrations a clear underestimation occurs, which is due to reabsorbed chlorophyll-a fluorescence (Heinz Walz GmbH, 2003). Therefore, samples of 500 µg L^-1^ or higher were diluted for adequate estimation of the chlorophyll-a concentration. Overall, the PHYTO-PAM gave good estimates of the chlorophyll-a concentration despite variations in the chlorophyll/fluorescence ratio may be common in field samples (Jakob et al., 2005).

**Figure S3**. Comparison of the chlorophyll-a concentration determined by hot ethanol extraction and by PHYTO-PAM based on in vivo fluorescence in 115 field samples.

In an experiment in which natural seston from an urban pond was incubated at three temperatures (20°C, 25°C, 30°C) with or without addition of nutrients (14 mg N L^-1^, 1.4 mg P L^-1^) cyanobacterial and eukaryote algae chlorophyll-a concentrations were measured using the PHYTO-PAM, while cell counts were performed microscopically using a Sedgwick Rafter counting chamber (Lürling et al., 2017). Cyanobacterial chlorophyll-a concentrations matched with cyanobacterial cell counts (log(CHLblue) = - 2.2129 × 0.8132 log(cells_cyanobacteria_), r^2^ = 0.927) and eukaryote chlorophyll-a concentrations were in good agreement with algal cell counts (log(CHLgreen+brown) = - 3.1680 × 1.1647 log(cells_eukaryote algae_ ), r^2^ = 0.965) (**Figure S4**).

**Figure S4**. Comparison of the cyanobacterial chlorophyll-a concentrations determined by PHYTO-PAM and microscopy counts of cyanobacteria (filled circles) and of eukaryote algal chlorophyll-a concentrations and algal cell counts (open circles).

**Figure S5.** The mean summer water temperature (ºC) in urban waters in The Netherlands during the period 2006-2012. The error bars indicate 1 SD. Twenty degrees is viewed as normal summer water temperature, while 25ºC is considered an extreme water temperature. During the heatwave year 2006, 32% of the sampled waters had a temperature of 25ºC or higher.

**Figure S6.** The TN:TP (upper panel) and DIN:SRP (lower panel) ratios in the water samples from 39 surface waters at start of the experiment used to identify N-limited and P-limited conditions.

**Table S1.** Overview of sampled waters, location and sampling date.

| Place/location | Latitude | Longitude | Name water body | Sampling date | | |
| --- | --- | --- | --- | --- | --- | --- |
| Asten | 51º24’18.38” | 5º44’53.50” | Vijver in Burg. Ploegmakerspark | | 16/07/2010 |  |
| Beek & Donk | 51º32’03.63” | 5º37’41.56” | Vijver aan Otterweg | | 16/07/2010 |  |
| Bennekom | 51º59’43.91” | 5º40’11.72” | Vijver aan Kierkamperweg | | 23/07/2010 |  |
| Bergen-op-Zoom | 51º29’51.19” | 4º17’40.46” | Vijver Anton van Duinkerkenpark | | 22/09/2010 |  |
| Bergen-op-Zoom | 51º30’37.40” | 4º17’32.10” | Kleine Melanen | | 22/09/2010 |  |
| Boxtel | 51º36’03.94” | 5º18’54.10” | Vijver aan Parkweg | | 11/09/2010 |  |
| Boxtel | 51º35’53.55” | 5º18’22.86” | Essche Heike | | 11/09/2010 |  |
| Breda | 51º36’00.15” | 4º46’56.12” | Vijver in Lienepark | | 22/07/2010 |  |
| Budel | 51º14’02.38” | 5º35’56.44” | Ringelsven | | 16/07/2010 |  |
| Deurne | 51º26’55.19” | 5º47’14.97” | Vijver aan Burgemeester Roefslaan | | 16/07/2010 |  |
| Ede (1) | 52º01’27.05” | 5º38’43.88” | Vijver aan Verenigde naties | | 23/07/2010 |  |
| Ede (2) | 52º02’03.84” | 5º38’56.83” | Vijver aan Jachtlaan | | 23/07/2010 |  |
| Ede (3) | 52º02’20.99” | 5º38’43.67” | Vijver bij Valkestein | | 23/07/2010 |  |
| Eindhoven | 51º29’26.70” | 5º28’33.72” | Stiffeliovijver | | 30/07/2010 |  |
| Etten-Leur | 51º34’09.41” | 4º39’02.74” | Vijver aan Vlaamse Schuur | | 22/09/2010 |  |
| Grave | 51º45’08.24” | 5º44’57.30” | Vijver aan Anna van Burenweg | | 06/09/2010 |  |
| Grave | 51º45’24.44” | 5º44’06.73” | Vijver G.W. Lovendaalsingel | | 06/09/2010 |  |
| Heesch | 51º44’09.00” | 5º32’24.65” | Vijver aan Langven | | 22/07/2010 |  |
| Heesch | 51º43’41.93” | 5º32’11.23” | Vijver De Ploeg | | 22/07/2010 |  |
| Maarheeze | 51º18’22.25” | 5º37’04.85” | Vijver aan Poelsnep | | 16/07/2010 |  |
| Ooltgensplaat | 51º41’01.14” | 4º22’02.35” | Volkerak-Zoommeer Buitendijk | | 30/07/2010 |  |
| Ooltgensplaat | 51º40’58.59” | 4º21’10.83” | Haven | | 30/07/2010 |  |
| Roosendaal | 51º30’41.82” | 4º26’42.02” | Vijver Dadelberg | | 22/09/2010 |  |
| Roosendaal | 51º30’49.33” | 4º26’43.82” | Vijver Dubbelberg | | 22/09/2010 |  |
| Roosendaal | 51º30’52.84” | 4º26’34.14” | Vijver Enclaveberg | | 22/09/2010 |  |
| Sint-Oedenrode | 51º33’58.54” | 5º27’44.56” | Molenwielvijver | | 30/07/2010 |  |
| Sint-Oedenrode | 51º34’27.41” | 5º26’58.30” | Visvijver in Park De Kienehoef | | 14/07/2010 |  |
| Sint-Oedenrode | 51º34’31.32” | 5º26’54.88” | Roeivijver in Park De Kienehoef | | 06/09/2010 |  |
| Someren | 51º22’54.31” | 5º42’21.23” | Vijver aan Wilbertshof | | 16/07/2010 |  |
| Son | 51º30’48.98” | 5º29’16.10” | Vijver aan Europalaan | | 16/07/2010 |  |
| Tilburg | 51º32’09.34” | 5º05’35.02” | Vijver aan de Berglandweg | | 11/09/2010 |  |
| Tilburg | 51º32’21.42” | 5º05’05.20” | Vijver aan de Kaukasusweg | | 11/09/2010 |  |
| Tilburg | 51º32’14.66” | 5º04’35.74” | Stappegoor | | 11/09/2010 |  |
| Tilburg | 51º32’57.18” | 5º02’58.38” | Reggevijver | | 11/09/2010 |  |
| Tilburg | 51º32’36.19” | 5º06’16.13” | EsscheStroom Vijver | | 11/09/2010 |  |
| Tilburg | 51º35’32.91” | 4º59’46.56” | Vijver Hoge Witsie | | 11/09/2010 |  |
| Valkenswaard | 51º20’19.88” | 5º28’05.84” | Dragonder | | 16/07/2010 |  |
| Wageningen | 51º58’07.98” | 5º40’38.33” | Dreyenvijver | | 22/07/2010 |  |
| Wageningen | 51º59’17.33” | 5º40’00.50” | Lumenvijver | | 14/07/2010 |  |

**Table S2**. Correlation coefficients (Pearson Product Moment Correlation ***r***) and corresponding *p* values for the water quality variables cyanobacteria chlorophyll-a (CHLcya), eukaryote algae chlorophyll-a (CHLeuk), total chlorophyll-a (CHLtot.), turbidity (NTU), electric conductivity (EC), pH, temperature (Temp), oxygen concentration (O_2_ conc), oxygen saturation (O2 %), total nitrogen (TN), total phosphorus (TP), phosphate (PO_4_-P), ammonium (NH_4_-N), and nitrate (NO_3_-N), Significant correlations (*p* < 0.05) are presented in bold.

| **r↓ ∕ *p*→** | **CHL_cya_** | **CHL_euk_** | **CHL_tot_** | **NTU** | **EC** | **pH** | **Temp** | **O_2_ conc** | **O_2_ %** | **TN** | **TP** | **PO_4_-P** | **NH_4_-N** | **NO_3_-N** |
| --- | --- | --- | --- | --- | --- | --- | --- | --- | --- | --- | --- | --- | --- | --- |
| **CHL_cya_** |  | 0.074 | **<0.001** | **<0.001** | 0.553 | 0.196 | 0.754 | 0.932 | 0.996 | 0.694 | 0.254 | 0.076 | **0.022** | 0.303 |
| **CHL_euk_** | -0.290 |  | 0.664 | 0.250 | 0.505 | 0.805 | 0.510 | 0.775 | 0.188 | 0.180 | 0.113 | 0.243 | 0.154 | 0.423 |
| **CHL_tot_** | **0.975** | -0.072 |  | **<0.001** | 0.875 | 0.160 | 0.861 | 0.578 | 0.706 | 0.916 | 0.410 | 0.117 | **0.042** | 0.206 |
| **NTU** | **0.877** | -0.191 | **0.869** |  | 0.755 | 0.084 | 0.949 | 0.387 | 0.904 | 0.809 | 0.334 | 0.078 | **0.018** | 0.198 |
| **EC** | -0.001 | -0.112 | -0.026 | -0.053 |  | 0.138 | **0.001** | 0.266 | 0.805 | 0.821 | 0.742 | 0.920 | 0.975 | 0.818 |
| **pH** | 0.214 | 0.041 | 0.232 | 0.288 | 0.245 |  | **<0.001** | 0.737 | **<0.001** | 0.057 | 0.534 | 0.375 | 0.245 | 0.552 |
| **Temp** | 0.052 | -0.110 | 0.029 | -0.011 | **0.509** | **0.515** |  | 0.347 | **0.043** | **0.043** | **0.043** | **0.022** | **0.027** | 0.175 |
| **O_2_ conc** | -0.115 | 0.055 | -0.108 | -0.167 | -0.214 | 0.065 | -0.181 |  | 0.058 | 0.612 | 0.772 | 0.889 | 0.728 | 0.880 |
| **O_2_ %** | 0.017 | 0.251 | 0.073 | -0.024 | 0.048 | **0.660** | **0.379** | 0.356 |  | 0.368 | 0.627 | 0.743 | 0.559 | 0.978 |
| **TN** | 0.065 | -0.219 | 0.017 | 0.041 | 0.038 | -0.311 | **-0.330** | -0.098 | -0.174 |  | **<0.001** | **<0.001** | **<0.001** | 0.900 |
| **TP** | 0.187 | -0.258 | 0.136 | 0.161 | 0.055 | -0.104 | **-0.330** | -0.056 | -0.094 | **0.727** |  | **<0.001** | **<0.001** | 0.599 |
| **PO_4_-P** | 0.287 | -0.191 | 0.255 | 0.289 | 0.017 | -0.148 | **-0.372** | -0.027 | -0.064 | **0.614** | **0.889** |  | **<0.001** | 0.594 |
| **NH_4_-N** | **0.365** | -0.233 | **0.327** | **0.382** | -0.005 | -0.193 | **-0.358** | -0.067 | -0.113 | **0.747** | **0.854** | **0.948** |  | 0.767 |
| **NO_3_-N** | 0.169 | 0.132 | 0.207 | 0.214 | 0.038 | -0.010 | -0.225 | -0.029 | -0.005 | -0.021 | -0.087 | 0.088 | 0.049 |  |

**Table S3**. Overview of measured water quality variables in the different surface waters; cyanobacteria chlorophyll-a, eukaryote algae chlorophyll-a, pH, oxygen concentration (O_2_ mg/l), oxygen saturation (O_2_ %), electric conductivity (EC), temperature (Temp), turbidity (NTU), Secchi depth (b indicates bottom sight), nitrate (NO_3_-N), phosphate (PO_4_-P), ammonium (NH_4_-N), total phosphorus (TP) and total nitrogen(TN) concentrations. --- indicates not measured.

|  |  |  | **CHLa conc**. | | | |  | |  | |  | |  | | |  | |  | | |  |  | | |  |
| --- | --- | --- | --- | --- | --- | --- | --- | --- | --- | --- | --- | --- | --- | --- | --- | --- | --- | --- | --- | --- | --- | --- | --- | --- | --- |
| Nr. | Location | **Cyano.**  **(µg/l)** | | **Eukar.**  **(µ/l)** | **pH**  **(-)** | **O2**  **(mg/l)** | | **O2**  **(%)** | | **EC**  **(µS/cm)** | | **Temp.**  **(ºC)** | | **NTU**  **(-)** | **Sd**  **(cm)** | | **NO_3_-N**  **(mg/l)** | | **PO_4_-P**  **(µg/l)** | **NH_4_-N**  **(mg/l)** | | | **TP**  **(mg/l)** | **TN**  **(mg/l)** | |
| 1 | Asten | 0.02 | | 3.56 | 7.59 | 3.7 | | 42 | | 572 | | 21.4 | | 2.33 | 100b | | 0.01 | | 3.08 | 0.01 | | | 0.14 | 1.40 | |
| 2 | Beek & Donk | 204.96 | | 18.55 | 9.12 | 17.6 | | 216 | | 517 | | 24.7 | | 70.9 | 23 | | 0.06 | | 5.30 | 0.03 | | | 0.13 | 0.83 | |
| 3 | Bennekom | 7.71 | | 79.18 | 7.67 | 7 | | 83 | | 525 | | 23.9 | | 9.95 | --- | | 0.00 | | 31.75 | 0.01 | | | 0.20 | 0.77 | |
| 4 | Bergen-op-Zoom | 1.00 | | 3.97 | 6.91 | 36 | | 31 | | 214 | | 15.7 | | 6.29 | 101b | | 0.00 | | 2.83 | 0.01 | | | 0.17 | 1.18 | |
| 5 | Bergen-op-Zoom | 6.76 | | 62.70 | 7.06 | 10 | | 84 | | 159 | | 15.6 | | 13.5 | 100b | | 0.01 | | 68.90 | 0.02 | | | 0.14 | 0.73 | |
| 6 | Boxtel | 83.30 | | 60.52 | 7.26 | --- | | --- | | 171 | | 16.6 | | 44.5 | 50 | | 0.17 | | 16.60 | 0.17 | | | 0.41 | 1.71 | |
| 7 | Boxtel | 54.08 | | 37.32 | 7.94 | --- | | --- | | 249 | | 16.1 | | 33.9 | --- | | 0.00 | | 47.23 | 0.01 | | | 0.15 | 0.79 | |
| 8 | Breda | 72.49 | | 43.53 | 9.2 | 17.1 | | 217 | | 356 | | 25.0 | | 21.8 | 64 | | 0.00 | | 0.00 | 0.00 | | | 0.10 | 0.98 | |
| 9 | Budel | 708.11 | | 0.10 | 8.71 | 4.8 | | 55 | | 391 | | 21.5 | | 116 | 25 | | 0.01 | | 101.80 | 0.09 | | | 0.37 | 0.56 | |
| 10 | Deurne | 104.46 | | 11.85 | 8.68 | 10.5 | | 120 | | 517 | | 24.7 | | 30.4 | 42 | | 0.00 | | 30.42 | 0.04 | | | 0.42 | 1.71 | |
| 11 | Ede (1) | 21.14 | | 102.31 | 8.76 | 11.7 | | 140 | | 556 | | 24.7 | | 14.5 | 50 | | 0.00 | | 12.20 | 0.01 | | | 0.22 | 1.16 | |
| 12 | Ede (2) | 5.35 | | 81.06 | 8.05 | 12.1 | | 147 | | 648 | | 24.9 | | 7.3 | --- | | 0.00 | | 72.50 | 0.01 | | | 0.26 | 1.24 | |
| 12 | Ede (3) | 14.05 | | 58.71 | 8.7 | 10.9 | | 132 | | 573 | | 24.8 | | 14.3 | --- | | 0.00 | | 18.25 | 0.02 | | | 0.19 | 1.69 | |
| 14 | Eindhoven | 81.11 | | 80.00 | 8.77 | 7.6 | | 86 | | 260 | | 21.1 | | 50.3 | 25 | | 0.13 | | 8.20 | 0.01 | | | 0.11 | 0.19 | |
| 15 | Etten-Leur | 2.76 | | 27.61 | 8.05 | 13.2 | | 113 | | 363 | | 15.5 | | 18.1 | 45 | | 0.14 | | 1102.44 | 2.01 | | | 1.40 | 2.34 | |
| 16 | Grave | 200.81 | | 96.86 | 7.44 | 9.1 | | 91 | | 459 | | 16.0 | | 68.6 | 25 | | 1.81 | | 175.10 | 0.22 | | | 0.08 | 1.34 | |
| 17 | Grave | 808.73 | | 0.00 | 7.97 | 8.3 | | 84 | | 417 | | 16.6 | | 209 | --- | | 0.11 | | 817.60 | 2.71 | | | 0.90 | 4.68 | |
| 18 | Heesch | 54.15 | | 39.17 | 7.96 | 8.9 | | 100 | | 248 | | 21.6 | | 22.7 | 30 | | 0.00 | | 20.39 | 0.01 | | | 0.09 | 0.01 | |
| 19 | Heesch | 0.00 | | 17.68 | 7.6 | 6.3 | | 73 | | 480 | | 22.9 | | 8.02 | 100b | | 0.05 | | 57.57 | 0.24 | | | 0.39 | 2.36 | |
| 20 | Maarheeze | 0.00 | | 14.45 | 7.4 | 2.9 | | 31 | | 358 | | 19.2 | | 37.5 | --- | | 0.01 | | 10.45 | 0.03 | | | 0.17 | 1.66 | |
| 21 | Ooltgensplaat | 5.89 | | 65.22 | 9.34 | --- | | --- | | 936 | | 23.1 | |  |  | | 0.01 | | 6.23 | 0.03 | | | 0.16 | 0.97 | |
| 22 | Ooltgensplaat | 0.28 | | 16.92 | 7.67 | 4.3 | | 51 | | 1070 | | 23.0 | | 8.72 | 75 | | 0.00 | | 14.31 | 0.00 | | | 0.28 | 1.04 | |
| 23 | Roosendaal | 1.03 | | 22.03 | 6.75 | 7.2 | | 60 | | 313 | | 14.4 | | 8.55 | 50 | | 0.08 | | 775.99 | 2.45 | | | 1.15 | 10.85 | |
| 24 | Roosendaal | 0.00 | | 40.77 | 7.05 | 10.9 | | 89 | | 379 | | 15.1 | | 10.3 | 50 | | 0.00 | | 4.25 | 0.01 | | | 0.37 | 3.13 | |
| 25 | Roosendaal | 43.43 | | 182.33 | 7.91 | 16.8 | | 139 | | 86 | | 15.6 | | 31.9 | 38 | | 0.04 | | 2.90 | 0.01 | | | 0.07 | 0.37 | |
| 26 | Sint-Oedenrode | 27.35 | | 107.20 | 7.85 | 6.9 | | 74 | | 473 | | 19.6 | | 33.7 | 45 | | 0.00 | | 8.40 | 0.01 | | | 0.17 | 1.13 | |
| 27 | Sint-Oedenrode | 289.57 | | 14.23 | 8.56 | 12 | | 147 | | 273 | | 26.1 | | 28.3 | 25 | | 0.01 | | 12.90 | 0.03 | | | 0.17 | 0.90 | |
| 28 | Sint-Oedenrode | 40.21 | | 77.35 | 8.93 | 11.3 | | 112 | | 196.5 | | 15.7 | | 27.5 | 40 | | 0.00 | | 10.36 | 0.03 | | | 0.65 | 3.37 | |
| 29 | Someren | 130.65 | | 57.51 | 7.19 | 2.8 | | 32 | | 227 | | 22.1 | | 58.1 | 40 | | 0.00 | | 12.00 | 0.06 | | | 0.22 | 1.17 | |
| 30 | Son | 175.60 | | 48.82 | 8.72 | 10.4 | | 120 | | 306 | | 22.2 | | 77.7 | 25 | | 0.26 | | 98.80 | 0.15 | | | 0.35 | 1.31 | |
| 31 | Tilburg | 22.62 | | 12.84 | 8.09 | --- | | --- | | 81 | | 16.4 | | 19.6 | 50 | | 0.00 | | 3.71 | 0.01 | | | 0.28 | 1.23 | |
| 32 | Tilburg | 4.35 | | 76.45 | 8.29 | --- | | --- | | 63 | | 16.7 | | 13.4 | -- | | 0.01 | | 25.94 | 0.18 | | | 0.15 | 1.27 | |
| 33 | Tilburg | 0.00 | | 3.88 | 7.62 | --- | | --- | | 196 | | 17.5 | | 3.12 | 100b | | 0.00 | | 0.94 | 0.01 | | | 0.17 | 1.40 | |
| 34 | Tilburg | 110.29 | | 70.07 | 7.83 | --- | | --- | | 69 | | 17.8 | | 47.3 | 35 | | 0.06 | | 45.10 | 0.03 | | | 0.10 | 0.33 | |
| 35 | Tilburg | 38.87 | | 53.80 | 7.01 | --- | | --- | | 77 | | 16.8 | | 20.8 | 45 | | 0.02 | | 50.80 | 0.05 | | | 0.06 | 0.37 | |
| 36 | Tilburg | 30.69 | | 74.66 | 8.43 | --- | | --- | | 278 | | 17.5 | | 23.6 | 58 | | 0.42 | | 204.40 | 0.37 | | | 0.27 | 0.32 | |
| 37 | Valkenswaard | 102.59 | | 12.02 | 9.38 | 7.3 | | 84 | | 201 | | 22.4 | | 107 | 25 | | 0.00 | | 3.60 | 0.01 | | | 0.15 | 0.33 | |
| 38 | Wageningen | 22.81 | | 49.53 | 7.12 | 5.2 | | 60 | | 101.2 | | 23.2 | | 30.4 | 100b | | 0.02 | | 10.03 | 0.29 | | | 0.20 | 1.64 | |
| 39 | Wageningen | 208.32 | | 22.56 | --- | --- | | --- | | --- | | --- | | 62.4 | --- | | 1.32 | | 11.00 | 0.12 | | | 0.11 | 1.16 | |
